# COVID-19 Deterred Career Path of Our Undergraduate Neuroscience Students: Educators’ Perspective

**DOI:** 10.1523/ENEURO.0384-22.2022

**Published:** 2022-10-04

**Authors:** Ami P. Raval, Helen M. Bramlett

**Affiliations:** 1 Department of Neurology, Peritz Scheinberg Cerebral Vascular Disease Research Laboratory, University of Miami, Miami, FL, 33136; 2 Department of Neurological Surgery, Leonard M. Miller School of Medicine, University of Miami, Miami, FL, 33136; 3 Bruce W. Carter Department of Veterans Affairs Medical Center, Miami, FL, 33136

**Keywords:** laboratory research training, postbaccalaureate, premedical tracks, shadowing, Zoom school

Almost every industry had a deer-in-the-headlights moment when the COVID-19 pandemic forced the world to a halt. The education field was faced with an unprecedented situation. How do we continue our instruction with this bizarre reality that we must accept as the new normal? Educators from kindergarten to university levels had to urgently adapt their ways to keep the mission of education alive. While some teaching methods remained effective despite remote learning and “Zoom school,” some were simply not possible to implement given social distancing and occupancy restriction guidelines at the time. As an undergraduate neuroscience educator, one daunting dilemma was how to continue laboratory research training of our young neuroscientists. Universities across the country had to send students home and restrict access to campuses. Many of our undergraduate neuroscience students are on premedical tracks, and these restrictions added much more complexity to their goals of meeting medical school requirements of shadowing physicians or getting volunteer positions in ongoing clinical research. These research training exposures are essential, not only for future medical or graduate school admissions, but more importantly these early training experiences help them to decide the career paths they want to pursue to continue their postbaccalaureate education. As their professors, we felt their panic as their whole plans to prepare themselves for graduate or medical schools flew from their grasp because of this global situation which was out of their control.

The task to involve undergraduates in much needed scientific research training is already difficult. The COVID-19 pandemic only compounded this difficulty as research laboratories and principal investigators (PIs) could not take on this additional responsibility because of (1) lockdowns and maximum occupancy limits restricting access to laboratories, (2) educators’ scramble to figure out the logistics of continuing with their teaching assignments taking them away from continuing the research training of undergraduate students, (3) students being sent home from universities during the early lockdown phase, and (4) strict testing, quarantining, and social distancing guidelines on clinical/research campuses. After we moved past the peaks of the pandemic lockdowns and access to vaccines increased, universities opened their doors to students again. However, PIs themselves were hard at work to regain their lost research time and once more undergraduate students were deprioritized and set aside.

After a two-year lapse in research training because of the pandemic, most undergraduate students had no choice but to apply to medical or graduate school for admission with their application falling short of research experience. Many were denied admission because of this missing vital part of their scientific education. As a result, many recently graduated students are searching desperately to find internships or paid research assistant positions to include research proficiency in their curricula vitae. In this process, they face barriers as they find that institutions are hesitant to hire them based on their lack of research experience, which is unfortunately ironic. The ones who can afford to are trying to apply to master’s programs hoping that these degree programs have research components where they could catch up on the years of potential research training they missed out on because of the pandemic.

The remaining pool of bright students with undergraduate degrees are turning back to their professors and asking whether these professors could accommodate them for any volunteer research position. Undergraduate neuroscience educators like us get bombarded by students for requests to join our laboratories for research experience, and for recommendations and referrals to join other laboratories in the same university, across the country, and even abroad. We have received countless requests from students who have graduated during the pandemic, but we also have responsibilities to mentor and involve students who are currently in our undergraduate programs that need the very training that these newly graduated students missed out on. This backlog of students requiring research is a problem that brings us great concern. We are both educators and active neuroscientists, and we want nothing more than to accommodate every student we come across in our labs for research training to foster the next generation of scientific excellence. Sadly, this is next to impossible given limited resources, physical space constraints of laboratories, and the sheer number of requests we are inundated with. Being forced to say “no” and let our own intelligent students go without the much-needed hands-on scientific training that they deserve is a harder task than one can imagine.

Since resources are limited, we had to be creative in devising ways in which we could involve as many students as possible so they could experience any sort of contact with the world of scientific research. Approaches like involving them in literature review or data analysis accommodated students who participated in our labs remotely. These approaches are also effective involving students who may be working a second job to make ends meet or those who moved back with family after graduating from their undergraduate institutions, so we have continued these practices even after social distancing and laboratory occupancy restrictions have been relaxed. In the laboratory, hands-on training can be achieved by staggering students in shifts to avoid overcrowding. This approach is another way to maximize the number of students getting valuable training, and helps us involve students from both the pool of current undergraduates that we train as well as the recently graduated students looking for opportunities.

There is a consensus about the need for more scientists with postgraduate education and training in all fields of science. A reason for this deficit is not the lack of interest in scientific career-paths by undergraduate students, but the limited number of opportunities they have available to participate and immerse themselves in real-world scientific research. Universities have tried to provide opportunities for students to participate in internships, volunteer positions, and short/long-term research programs as a means to promote the scientific field to students to meet this need for more career researchers. Unfortunately, without adequate financial support for these programs, students are unable to participate in these research opportunities because of their need to seek other nonresearch-based summer employment. These jobs may provide financial supplements for school costs but do not provide intellectual growth or aid in their goals of attending a postgraduate research program. Consequently, most of the undergraduate students in biological disciplines lack “real” scientific experience during their school years. Students often get discouraged and rarely think about continuing their education once they graduate. Although there are multiple programs including those such as the National Institutes of Health and American Heart Association that provide support for the training of undergraduate students, these types of special fellowships and grants are few and far between. More investment needs to be made in our next generation of prospective neuroscientists and scientific researchers in general.

One thing for sure is that the COVID-19 pandemic reminded us that science matters. All over the world, countries have turned to their scientific community for advice, solutions, and hope for a future beyond COVID-19. Now, more than ever, we must focus on training our students to become effective scientific and clinical researchers.

